# Feeding Intolerance and Poor Growth in Infants with Gastroschisis: Longitudinal Experience with Consecutive Patients over Thirteen Years

**Published:** 2015-10-01

**Authors:** Saloni Balgi, Sarita Singhal, Georgia Mueller, Beau Batton

**Affiliations:** 1 Department of Pediatrics, Southern Illinois University School of Medicine, Springfield, Illinois; 2 Department of Pediatrics, University of Buffalo School of Medicine, Buffalo, New York; 3 Statistics & Epidemiology, Southern Illinois University School of Medicine, Springfield, Illinois

**Keywords:** Gastroschisis, Growth, Enteral nutrition, Preterm infant

## Abstract

**Objectives:** 1) To investigate in-hospital factors associated with delayed tolerance of full volume enteral nutrition and 2) To assess longitudinal growth in a contemporary population of infants with gastroschisis.

**Design:** Retrospective single-center study of all infants with gastroschisis

**Setting:** Level III neonatal intensive care unit in a free-standing Children’s Hospital

**Duration:** 13.5 years

**Materials & Methods:** Detailed data regarding demographics, nutritional support, growth, and infant outcomes was collected for all infants with gastroschisis. Linear regression was used to investigate in-hospital factors associated with feeding intolerance and poor growth.

**Results:** For 52 infants, the median gestational age at birth was 36 weeks, the median postnatal age to achieve full feeds was 22 days, and median in-hospital weight gain was 18 gm/day. With linear regression, there was a positive association between time to full feeds and both hospital length of stay (adjusted R2=0.503, p < 0.0001) and (unexpectedly) in-hospital weight gain (adjusted R2=0.125, p=0.0248). There was a negative association between in-hospital weight gain and preterm birth (adjusted R2=0.125, p=0.0356). For infants with longitudinal growth data, 35% had a weight < 5th percentile (of whom 67% were preterm).

**Conclusions:** Many infants with gastroschisis have poor growth before and after hospital discharge. Aggressive feeding advancement may be a contributing factor to this finding and preterm infants may be at greater risk for poor growth than term infants.

## INTRODUCTION

Gastroschisis is a rare congenital disease characterized by a full-thickness paraumbilical abdominal wall defect associated with evisceration of intra abdominal organs. The frequency of this anomaly appears to be increasing with a current incidence of four to five per 10,000 live births [1-3]. Since contemporary survival rates exceed 90% [4], disease management has shifted to optimizing surgical outcomes and minimizing the impact of adverse events such as intestinal ischemia, short gut syndrome, and nosocomial infection.

Studies of gastroschisis typically focus on the surgical approach to care and associated complications [5-7]. There is limited information available on optimizing enteral nutrition or maximizing postnatal growth in infants with gastroschisis [8-10]. It is not clear which factors, if any, increase the risk for prolonged feeding intolerance or poor growth following abdominal wall closure [11]. Although a diagnosis of intrauterine growth restriction is common in such fetuses, this finding has not been associated with worse infant outcomes [12-14]. The impact of maternal variables, preterm birth, and other infant variables on feeding tolerance and postnatal growth also remains unclear. 

A better understanding of risk factors for suboptimal nutrition and growth may assist with improving patient management strategies for infants with gastroschisis. The objectives of this study were to investigate in-hospital factors associated with delayed tolerance of full volume enteral nutrition and to assess longitudinal growth in a contemporary population of infants with gastroschisis.

## MATERIALS AND METHODS

This was a retrospective study of all infants with gastroschisis cared for in the 45 bed level III neonatal intensive care unit of St. John’s Children’s Hospital between January 1, 2000 and June 30, 2013. Hospital records were reviewed in detail for data extraction regarding maternal and infant demographics, in-hospital morbidities, growth prior to discharge, duration of hyperalimentation, hospital length of stay, surgical management of gastroschisis, and history of enteral feeds (timing of initiation of feeds, rate of advancement, and time to reach full volume feeds). Infants were routinely seen within one week of hospital discharge, at six months of age and at least annually beginning at one year. Weight, length, and head circumference at one and two years were obtained from the primary care physician’s office when available. The primary outcomes were the postnatal age at which full feeds (150 ml/kg/day) were reached and average in-hospital weight gain (grams/day). Secondary outcomes included the postnatal age at which enteral feeds were initiated, the rate feeds were advanced, duration of parenteral nutrition, and growth over the first two years. During this time, Cesarean delivery was not performed solely for gastroschisis and the surgical approach was consistent: when feasible a silo was placed shortly after birth followed by primary closure of the abdominal defect later in the first postnatal week. 

Data was analyzed using SPSS software, version 9.1.3 (Cary, North Carolina). The non-parametric Wilcoxon-Mann-Whitney t-test was used for continuous variables because the data did not follow a normal distribution. The normality assumption was tested with the Shapiro-Wilk test. Fisher Exact tests were used for two by two comparisons of categorical variables and the Chi-squared test of independence was used for maternal education. Linear regression models incorporating the following variables were developed to investigate factors influencing hospital length of stay, time to full feeds, and in-hospital weight gain: year of birth, gestational age (GA) at birth (preterm versus term), infant size (small versus appropriate for GA), presence or absence of intrauterine growth restriction, and mode of delivery. Diagnostic tests were performed for all linear models and no violations of assumptions were found after the log transformation of dependent variables hospital length of stay and tolerance of full feeds. This investigation was approved by the local Institutional Review Board with a waiver of informed consent.

## RESULTS

There were 53 infants with gastroschisis identified during the 13.5 year study period, including 33 (62%) preterm infants. One infant who died shortly after birth at 23 weeks GA was not included in subsequent analyses. Maternal and infant demographic data and infant in-hospital outcome data for the remaining 52 infants are presented in table 1. Data regarding surgical management, enteral nutrition, and in-hospital growth are reported in table 2. Abdominal wall closure occurred in the first postnatal week for 51 (98%) infants. Surgery was delayed until postnatal day 11 in a preterm infant born at 29 weeks GA. Seven (13%) infants developed hyperalimentation related cholestasis (direct bilirubin level >2 mg/dl). Short gut syndrome was diagnosed in six (12%) infants.

**Figure:**
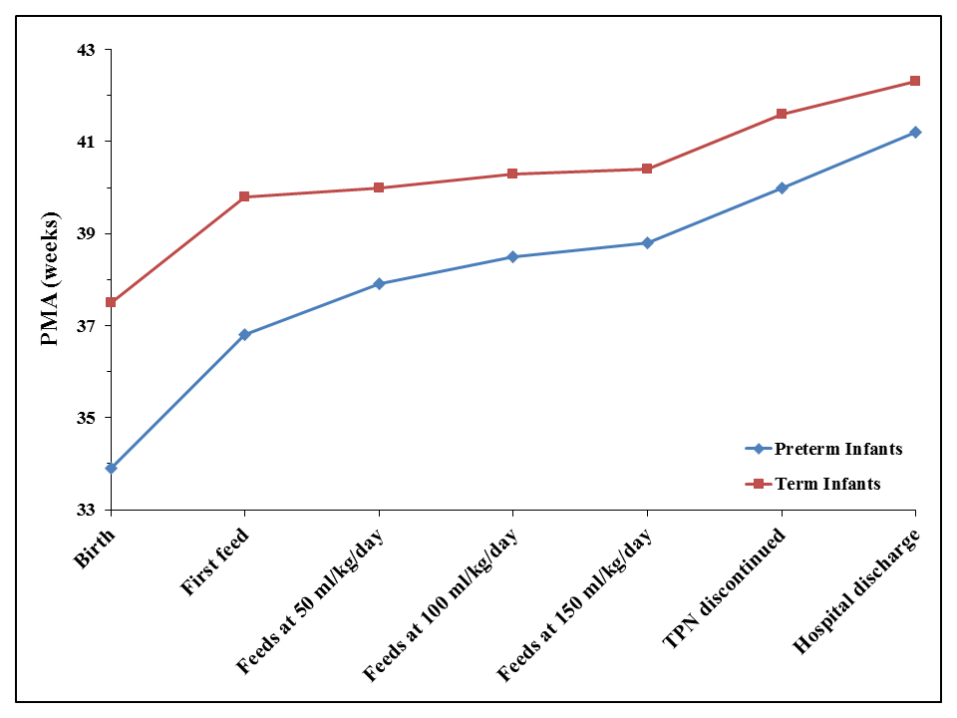
Figure 1: Median postmenstrual age (in weeks) of feeding achievements for term and preterm infants with gastroschisis.

Demographic data was similar for preterm and term infants as there was not a significant difference between groups in maternal self-reported age (p=0.5566), race (p=1.00), level of education (p=0.4185), smoking (p=0.5322), or type of insurance (p=0.7458). Mode of delivery (p=0.3819), infant gender (p=1.00), one and five minute Apgar score (p=0.8594 and p=0.9107, respectively), small for gestational age status (p=0.7659), and incidence of intrauterine growth restriction (p=0.1719) were also similar for preterm and term infants. There were no statistically significant differences in the timing of initiation of feeds, rate of feeding advancement, or time to tolerance of full volume feeds between preterm and term infants (table 2, figure 1) or in the hospital length of stay (p=0.1345). There was no correlation between the GA at birth and the postmenstrual age at hospital discharge (figure 2, p=0.0569).

**Figure:**
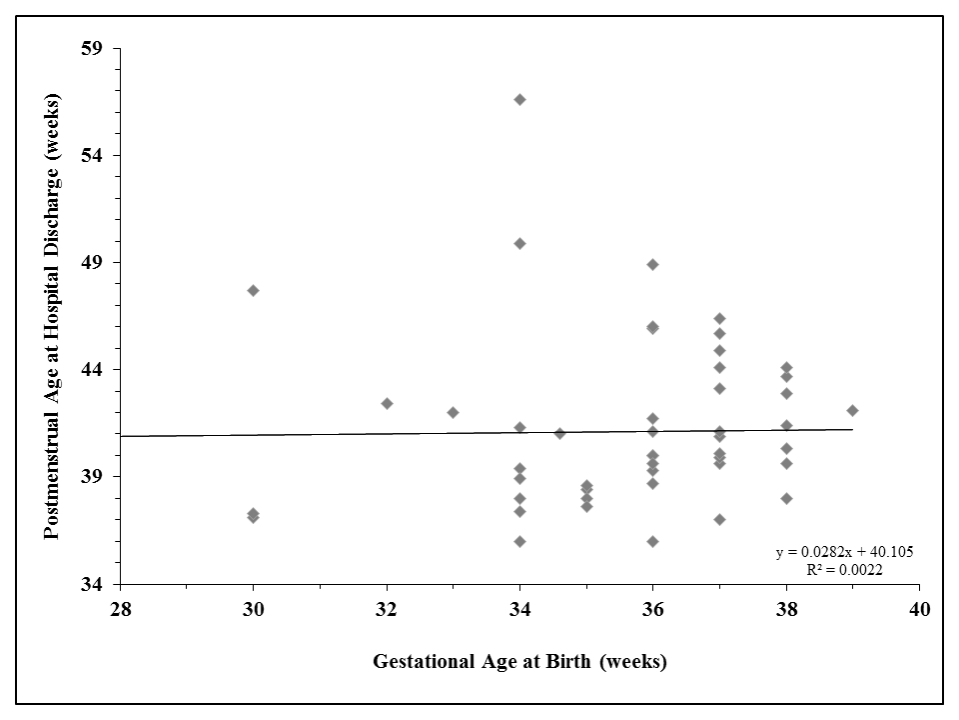
Figure 2: Relationship between gestational age at birth and postmenstrual age at initial hospital discharge for infants with gastroschisis.

**Figure:**
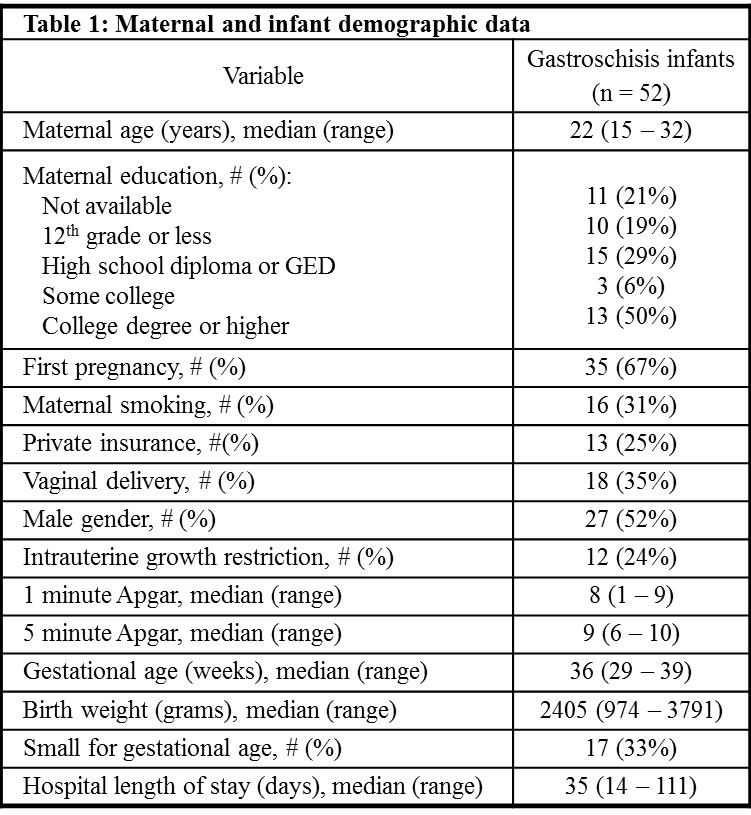
Table 1: Maternal and Infant Demographic Data

**Figure:**
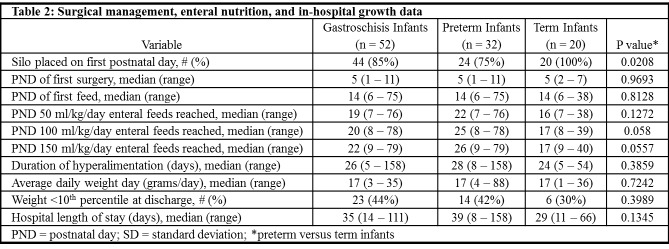
Table 2: Surgical Managament, Enteral nutrition, and in-hospital growth data

With linear regression, there was a significant positive association between time to full feeds and both hospital length of stay (adjusted R2=0.503, p < 0.0001) and in-hospital weight gain (adjusted R2=0.125, p=0.0248). In addition, there was a significant negative association between in-hospital weight gain and preterm birth (adjusted R2=0.125, p=0.0356) such that preterm infants had significantly lower weight gain than term infants. Year of birth, GA at birth, infant size, intrauterine growth restriction, and mode of delivery were not significant predictors of time to full feeds or any additional secondary outcomes, including postnatal age at which enteral feeds were initiated, time to full feeds, rate of feeding advancement, duration of parenteral nutrition, or growth in the first two years (p>0.05 for all analyses). Longitudinal growth data at one to two years of age was available for 26infants. Eighteen (69%) of these infants were preterm. At latest follow-up, nine (35%) infants had a weight at or below the fifth percentile, including six (23%) preterm infants. Three preterm infants also had a length and head circumference at or below the fifth percentile.

## DISCUSSION

In this study, prolonged feeding intolerance requiring hyperalimentation was common in both preterm and term infants with gastroschisis. As expected, there was also a significant association between time to achieve full feeds and hospital length of stay. There were not any specific risk factors for prolonged feeding intolerance identified suggesting the duration of hyperalimentation and hospitalization cannot be accurately predicted prior to initiation of enteral nutrition.

Sub-optimal weight gain was common in infants with gastroschisis – 44% had a weight below the tenth percentile at hospital discharge. Potential explanations for this include inadequate parenteral nutrition, altered intestinal absorption of nutrients, and sub-optimal transition from parenteral to enteral nutrition. The limitations of neonatal parenteral nutrition are well recognized [15]. These limitations may be even more pronounced in gastroschisis infants because of a higher incidence of intrauterine growth restriction, increased caloric demands following surgery, and a significant likelihood of parenteral nutrition related liver disease [16, 17]. 

Unexpectedly, a longer time to full feeds was associated with better in-hospital weight gain. Sub-optimal nutrient intake associated with aggressive feeding advancement may contribute to poor weight gain. Optimizing the transition from parenteral to enteral nutrition has not been closely investigated for gastroschisis infants [18-21]. Enteral feeds are usually initiated at a low volume and gradually advanced if tolerated. Feeding tolerance is broadly defined, but often includes a re-assuring abdominal exam, regular stooling pattern, absence of significant emesis, and minimal feeding residuals. There are no strict criteria for advancing feeds or assessing feeding tolerance and practice varies substantially [20, 21]. Hyperalimentation is typically discontinued when an infant appears to be tolerating ~120 ml/kg/day of enteral nutrition. Although this volume may be sufficient to avoid serious dehydration or a catabolic state, it may not provide sufficient caloric intake for adequate growth. Thus, the transition from parenteral to enteral nutrition can lead to an extended period of insufficient caloric and nutrient intake which contributes to the poor growth observed in these infants [16, 17, 19, 22]. After discharge, surveillance of nutrition and growth is often sub-optimal [18, 19, 22]. In the current study, gastroschisis infants were not regularly followed by a dietitian following hospital discharge until 2009. It is likely that closer scrutiny of periodic weight gain and caloric intake will decrease the number of infants lost to follow-up and may improve longitudinal growth; however, there was insufficient data to examine this. 

Most outcomes were similar between preterm and term infants. As noted in the first figure, the typical postnatal course for preterm infants with gastroschisis paralleled that of term infants with preterm infants discharged at a lower median postmenstrual age. The only notable difference in outcomes was in longitudinal growth. With linear regression, preterm birth was associated with worse in-hospital weight gain and a disproportionate number of infants with poor growth at one to two years were preterm. Even without gastroschisis, poor postnatal growth is more common in preterm infants than term infants. The combination of prematurity and gastroschisis appears to have a negative synergistic effect on infant growth [16, 17]. Although not observed in the current study, a greater risk of adverse outcomes in preterm versus term infants with gastroschisis has been reported [16, 17]. Other factors negatively impacting growth and development may be more prevalent in preterm infants as well [23].

Strengths of this study include longitudinal data collected on consecutive patients for more than thirteen years, scrupulous detailed collection of feeding data, consistent approach to surgical closure, and consistent medical management of infants with gastroschisis. Study limitations include the limited number of infants with Short Gut Syndrome (which may have impacted hospital length of stay and weight gain), incomplete growth data at one and two years, and the retrospective nature of the study.

Poor growth is common for infants with gastroschisis. Preterm infants appear to be at greater risk for this than term infants both before and after the initial hospital discharge. Aggressive advancement of feeds may contribute to poor in-hospital growth and it is possible introducing feeds a slower rate or delaying the discontinuation of parenteral nutrition will improve postnatal growth. Further investigation is needed to investigate the potential benefits of slower feeding advancement and the potential risks, including more days with a central line, risk of associated liver disease, and longer hospital stay.

## Footnotes

**Source of Support:** None

**Conflict of Interest:** None
